# Ten simple rules for open human health research

**DOI:** 10.1371/journal.pcbi.1007846

**Published:** 2020-09-03

**Authors:** Aïda Bafeta, Jason Bobe, Jon Clucas, Pattie Pramila Gonsalves, Célya Gruson-Daniel, Kathy L. Hudson, Arno Klein, Anirudh Krishnakumar, Anna McCollister-Slipp, Ariel B. Lindner, Dusan Misevic, John A. Naslund, Camille Nebeker, Aki Nikolaidis, Irene Pasquetto, Gabriela Sanchez, Matthieu Schapira, Tohar Scheininger, Félix Schoeller, Anibal Sólon Heinsfeld, François Taddei

**Affiliations:** 1 Center for Research and Interdisciplinarity (CRI), Université de Paris, INSERM U1284, Paris, France; 2 Institute for Next Generation Healthcare, New York, New York, United States of America; 3 MATTER Lab, Child Mind Institute, New York, New York, United States of America; 4 Sangath, New Delhi, India; 5 COSTECH, Université de Technologie de Compiègne, Compiègne, France; LabCMO, Université du Québec à Montréal, Université Laval, Montreal, Canada; 6 Hudson Works LLC, Washington, District of Columbia, United States of America; 7 Four Lights Consulting LLC, Washington, District of Columbia, United States of America; 8 Department of Global Health and Social Medicine, Harvard Medical School, Boston, Massachusetts, United States of America; 9 Department of Family Medicine and Public Health, School of Medicine, University of California San Diego, San Diego, California, United States of America; 10 Center for the Developing Brain, Child Mind Institute, New York, New York, United States of America; 11 Harvard Kennedy School, Harvard University, Cambridge, Massachusetts, United States of America; 12 University of Geneva, Geneva, Switzerland; 13 Structural Genomics Consortium and Department of Pharmacology & Toxicology, University of Toronto, Toronto, Canada; 14 Healthy Brain Network, Child Mind Institute, New York, New York, United States of America; Dassault Systemes BIOVIA, UNITED STATES

## Introduction

We are witnessing a dramatic transformation in the way we do science. In recent years, significant flaws with existing scientific methods have come to light, including a lack of transparency, insufficient stakeholder involvement, disconnection from the public, and limited reproducibility of research findings [1–7]. These concerns have sparked the global Open Science movement, which seeks to revolutionize the practice of science. This new approach to science extends principles of openness to the entire research cycle, from hypothesis generation to data collection, analysis, interpretation, and dissemination. Open Science seeks to remove all barriers to conducting high quality, rigorous, and impactful scientific research by ensuring that the data, methods, and opportunities for collaboration are open to all. Emerging digital technologies and "big data" (see "Ten simple rules for responsible big data research" [8]) have further accelerated the Open Science movement by affording new approaches to data sharing, connecting researcher networks, and facilitating the dissemination of research findings.

Open scientific practices are also having a profound impact on the health sciences and medical research and, specifically, how we conduct clinical research with human participants. Human health research necessitates careful considerations for practicing science in an ethical manner. Given the particular urgency of human health research, a discipline with direct implications for people's health and wellbeing, doing good science takes on a different meaning than simply doing science well. It also requires the scientist to reassess the conventional view of human health research as a pursuit conducted by scientists on human subjects, and lays a greater emphasis on inclusive and ethical practices to ensure that the research takes into account the interests of those who would be most impacted by the research. Openness in the context of human health research comes with risks, raising concerns about privacy and security. However, openness also presents opportunities for people, including participants of research studies, to contribute in every capacity. At the core of open health research, scientific discoveries are not only the product of collaboration across disciplines, but must also be owned by the community that is inclusive of researchers, health workers, and patients and their families. To guide successful open health research practices, it is essential to carefully consider and delineate its guiding principles.

**Fig 1:**
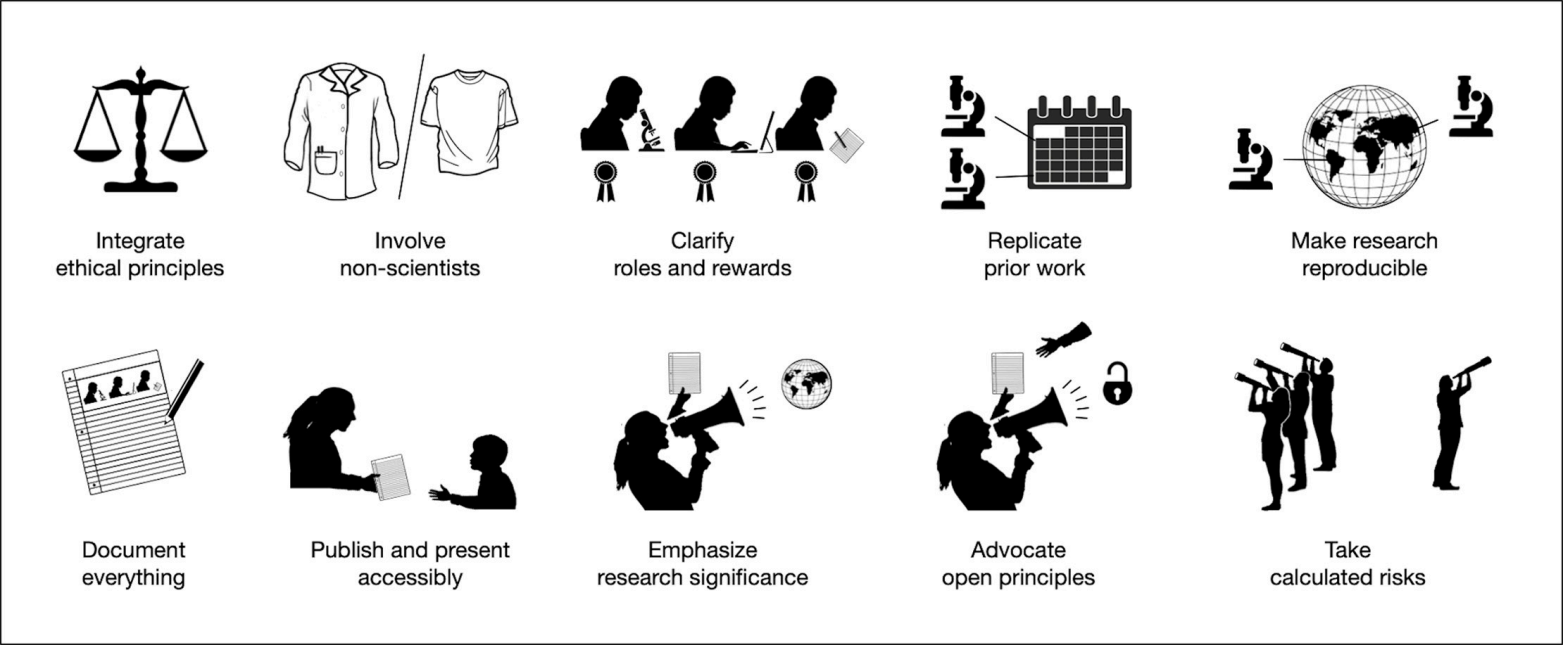
Our 10 simple rules for open human health research, presented graphically. Image sources: [147–156].

This Editorial is aimed at individuals participating in health science in any capacity, including but not limited to people living with medical conditions, health professionals, study participants, and researchers spanning all types of disciplines. We present ten simple rules (see Fig 1) that, while not comprehensive, offer guidance for conducting health research with human participants in an open, ethical, and rigorous manner. Implementing these rules can be difficult and resource intensive, and the rules can, at times. overlap with one another as well as conflict with one another. They present a challenge and may not be implemented all at once, but they are intended to accelerate and improve the quality of human health research. Work that fails to follow these rules is not necessarily poor quality research [9], especially if the reasons for breaking the rules are carefully considered and openly articulated (see Rule 6: document everything). While most of the responsibility of following these rules falls on researchers, anyone involved in human health research in any capacity [10] can apply them.

For each rule, we provide a very brief background motivating inclusion of the rule, followed by a few recommendations.

## Rule 1: Integrate ethical principles

Health research is no longer exclusive to scholars or medical professionals. Technology developers are increasingly engaging in and leading biomedical research, participants are taking on a more active role as partners in research, and nonscientists are even designing and deploying their own health research projects [11]. While this greater involvement of nontraditional parties in health research has the potential to advance the research in novel ways, it is critical for these parties to abide by ethical and responsible practices, to ensure privacy and safety. The tech industry continues to generate increasingly sophisticated digital technologies, such as wearable devices, mobile phone apps, and social media that can record more and more aspects of daily life [12–15] without direct, voluntary consent or clear information about how data will be used, shared, or reported. This can lead to unintended consequences, such as inappropriate disclosure of personal information and the spread of inaccurate or misleading information.

In a rapidly changing research landscape with shifting roles, it is crucial to emphasize and enforce core ethical principles, including respect for persons, justice and beneficence (doing what is right) [16], and respect for law and the public interest [17]. The well-established, tried and tested rules and regulations for behavioral and biomedical research involving human participants [18] must demonstrate voluntary participation via informed consent [19–22], perform risk assessment to determine if the probability and magnitude of potential harms are balanced against potential benefits, include those who may benefit most from knowledge gained, consider downstream societal implications, conduct an external review of study procedures before initiating any project, and develop additional protections for vulnerable stakeholders. We consider stakeholders broadly as any entity with the potential to be affected, directly or indirectly, by the project in question.

Include people on your team who bring expertise in research ethics, methods, and data management. This is especially important for successfully guiding open human health research, in which efforts to mitigate risks for human participants and uphold key ethical principles must be kept open and transparent. Carefully choose what data to collect and how to represent and store those data, remembering that while data storage costs have rapidly shrunk, other costs, including but not limited to compromised privacy and unauthorized access, are inherent in any data collection [23–26].

Take responsibility for building a peer review process into each study design with periodic checks and balances. Do not simply delegate consideration of ethical and responsible research practices solely to research ethics boards (also known as institutional review boards [IRBs]). The Connected and Open Research Ethics (CORE) initiative [27] is a global community interested in collaborating to shape ethical practices in digital research, and their resource library contains shared IRB-approved protocols and a forum for sharing expertise and answering questions. The Citizen Science Association has also developed and shared materials for conducting an IRB review [28], to help build an ethics review process for the citizen science community. Further resources to advance our understanding of the ethical, legal, and social implications of this emerging digital research ecosystem are provided by the CORE initiative [29,30], MobileELSI [31], Pervasive Data Ethics for Computational Research (PERVADE) [32], and Clinical Trials Transformation Initiative (CTTI) [33].

## Rule 2: Involve nonscientists

There are many roles nonscientists can take to advance human health research, beyond participation in traditional, computer task- or game-based citizen science projects [34,35]. First and foremost are the patients who are best served by the research, who can not only enroll as participants but also help define problems, goals, and measures of success. Any interested party, including patients, clinicians, ethicists, policy makers, funding agencies, and individuals from the general public [36], can and should partner with the research community at the different stages of research—soliciting ideas for funding, designing or coordinating studies, recruiting participants, collecting or analyzing data, interpreting or broadcasting results, participating in the peer review process [37–39], and so on. The website for the Office of Research Integrity provides a resource for learning about responsible scientific methods, the Basic Research Concepts [40].

Include nonscientist stakeholders throughout the scientific process in meaningful, informative, accessible, and engaging ways. From the very inception of a study, encourage and support the active participation of patients and other interested parties in defining research questions. Patient-led innovation platforms and patient-driven networks in health, such as PatientsLikeMe [41], help connect people suffering from common diseases to share their experiences and have spawned scientific studies [42]. When technologies are involved, collaborate with technology developers and end-users to ensure products are scientifically validated, evidence-based, and user-friendly.

For community-facing projects, hold meetings with community members to allow for concerns and questions to be voiced and responded to. Seek out opportunities to bridge divides among communities and their access to resources. For example, work to match stakeholder ideas and needs to other stakeholders’ skills and resources. Make efforts to raise awareness of complementary literatures and overcome disciplinary divides. Participate in funding opportunities for projects that involve non-research stakeholders and patient-centered outcomes, such as from the Patient-Centered Outcomes Research Institute (PCORI) [43].

Invite nonscientist stakeholders to take part in scientific events, such as conferences, seminars, workshops, and lab meetings [44,45]. Participate in such events as a non-scientist in research outside of your areas of expertise (e.g., stepping outside of your “comfort zone”). Actively engage with nonscientists and participants outside one's discipline; listen to, respect, and value their perspectives and opinions. Strive to engage a diverse population (e.g., demographic, gender representation, employment, education, etc.). Such diversity will ensure a better informed approach to the research, a greater interest in the research results, and broader generalizability of the research findings. This is especially important because the views and perspectives of patient groups who stand to benefit most from research are rarely considered or acknowledged, representing a persistent challenge across many areas of health research.

## Rule 3: Clarify roles and rewards

There are obvious benefits to clearly articulating what roles different contributors will play in a given research study and how they will be acknowledged or rewarded accordingly. Not only does it set up reasonable expectations for all parties, but it also avoids conflicts and misunderstandings commonly found in the academic research community related to authorship and allocation of funds and other resources [46]. Human health research raises the stakes considerably, given that it involves human participants, who are rarely acknowledged for their participation. Open human health research raises the bar further, as it engages many different stakeholders and increases the number of potential contributors who should be rewarded for their contributions.

Rewards in research for nonscientists, aside from the satisfaction of having contributed to science and possible monetary compensation or prizes [47–50], typically include information about their health or access to experimental treatment. Rewards for scientists are also often driven by forces beyond the individual’s control, such as funding, promotion, and tenure. While individual scientists do write proposals to request resources and support, it is rare for them to take a more hands-on approach and launch a crowdsourcing campaign, and many are apathetic to self-promotion through social media. We therefore focus our recommendations, for both scientists and nonscientists, on different forms of recognition as means of conferring and receiving rewards, rather than direct monetary or career gains.

At the outset of a research project, clarify contributor roles, acknowledgments, rewards, and code of conduct, e.g., see Conference Code of Conduct [51]. Use resources like "Ten Simple Rules for a Successful Collaboration" [52] and *Collaboration and Team Science*: *A Field Guide* [53] for guidance defining these roles. Also clarify when data or software can be released, how it will be released (e.g., Github, Figshare, Google Drive), and cite the resources you use [54]. Think beyond the usual contributor acknowledgments of "author," "editor," "contributor," "acknowledgment," etc. [55] and reconsider author order. In other words, clearly define and state what contributions would lead to what acknowledgments or rewards [56]. The International Committee of Medical Journal Editors provides guidance (the Vancouver Recommendations) that many journals require for submissions and are good practices to follow regardless of publisher requirements [57,58]. The Committee on Publication Ethics also provides hundreds of guiding documents, including flowcharts, specifically relating to authorship and contributorship [59–61].

Even outside of your own research, acknowledge where good, open, ethical, inclusive human health research practices are conducted. Be especially mindful to acknowledge open practices [62–64], research in languages in addition to English [65], and research from nontraditional actors [66]. Point out where greater efforts could be made toward better scientific practices. Lead by example, but also, when attending another’s talk or lecture, do ask for clarification on who contributed what, so as to encourage this practice in others.

Engage in more quantitative approaches of acknowledgment and reward. For example, rigorously quantify the degree to which your research and contributed or adopted resources that you use embrace openness, ethical practices, inclusiveness, etc. Think carefully about what "impact" means in relation to your work. For example, rather than (or in addition to) tracking academic citations, you may be more interested in fostering collaboration between particular previously siloed, isolated bodies of knowledge or in tracking some aspect of your research into practice. Make use of indicators that measure or estimate those types of impact [6,67–73].

## Rule 4: Replicate prior work

It is incumbent on researchers to ground their research in the context of prior work. The first step is often to confirm prior work by reproducing past results (apply the same methods to the same data to get the same results). To ensure that this prior work translates to a new study population or reimplementation of old methods, a researcher tries to corroborate prior work by replicating past results (collect new data, apply similar methods, to get similar results). Replication in science is presently in a woeful but improving state [74,75]. Science is by its nature uncertain, improving and replacing current models with better models over time. Replicating prior work helps to reduce this uncertainty and increase our confidence in the findings [76–79]. Conversely, past work can be reassessed in light of new findings as well [80], and past data collected by others can be independently reused or integrated with newer datasets [81,82].

Replication does not necessarily mean running a past study or its analysis again in exactly the same way—this may be a waste of resources if the original study was conducted on a small, nonrepresentative population using outdated approaches. Instead, use best available practices and sufficiently powered sample sizes from relevant populations to evaluate the state of knowledge and establish a sound foundation for a research program. Some conferences, such as the Organization for Human Brain Mapping, have given replication awards to encourage such studies [83].

Designate some of your time and research efforts to replication and confirmatory studies. Find prior work related to your research questions. Carry out replication studies by following published methods with new or existing open data, explaining your deliberate data acquisition choices [82]. Be mindful of the fact that validity and replicability are different, and that the goal of replication is to test validity or generalizability of the models in question [80]. Perform complementary analyses on published open data to further explore the data behind published findings [44].

## Rule 5: Make research reproducible

Just as it is crucial to try to replicate prior work to ground current research, it is likewise crucial to make your own research work reproducible as a foundation for future research. While replicability is the ability of a method to be repeated to obtain a consistent result, reproducibility is the extent to which the same conclusions can be drawn from the same data by using either the same or different methods. Data and methods must be subjected to scrutiny and evaluated for robustness and generalizability. This practice is not an act of generosity—if you do not make your data and methods available and clear to others, you undermine the credibility of your work and hinder the advance of science.

Follow FAIR (findable, accessible, interoperable, and reusable) principles in your scientific practices [84,85]. The following two rules regarding documentation and accessible presentation are most closely related to reproducibility. Specifically, for documentation to aid reproducibility it must be shared, just like presentations, and shared in formats (languages, descriptions, file types) that are easily accessible [86]. In practice, "data […] exist in small units, are linked to many other related units, and are difficult to interpret without considerable documentation and context" [54]. Adequate data documentation can be difficult and resource intensive [82,87]; while inadequate data management can severely compromise the scientific value and interpretability of the associated research. See "Ten Simple Rules for the Care and Feeding of Scientific Data" [88] for guidance.

Share data, methods, and documentation in open-access repositories [88]. At the very least, this practice enables consumers of your research to scrutinize your work. More importantly, other methods can be applied to your data, and your methods can be applied to other data, to test assumptions, hypotheses, methods, as well as data quality and generalizability. Digital containers (e.g., Docker and Singularity) make it much easier to conduct reproducible research within self-contained environments and help mitigate concerns about maintaining software and dependencies in different computing environments.

Since data breaches are a persistent challenge, provide participants with clear and accessible information about how data will be collected, stored, shared, and used in the future, while making it clear that no one can provide absolute guarantees about future data security. Do not collect identifiable information if you do not need it for your research. Otherwise, separate identifiable from currently nonidentifiable information and, if possible, destroy the identifiable information at the conclusion of the study. Be sure to scrub software and other documentation of any references to participant-specific information. Apply best practices for data deidentification, such as mixing data or adding noise to data (differential privacy, face removal from images, etc.). In cases where data cannot be made fully open, deposit metadata-only records in a repository with instructions for who can gain access to the data and how. There is a variety of options available when choosing a data repository to store and share data and metadata, such as Open Science Framework (osf.io), Zenodo (zenodo.org), Synapse (synapse.org), Dryad (datadryad.org), and Harvard Dataverse (dataverse.harvard.edu). Directories of data repositories include re3data (re3data.org) and OpenDOAR (v2.sherpa.ac.uk/opendoar).

In your published methods and results, be as clear as you can about your assumptions, hypotheses, measures, and methods. Summary statistics and thresholds can be useful, but commonly-reported statistics such as *p* values are not one-size-fits-all measures of research quality or reproducibility [89–91]. Where word limits or other constraints prevent adequate articulation for clarity, publish as supplementary information elsewhere (see Rule 6: document everything and Rule 7: publish and present accessibly).

## Rule 6: Document everything

In service of the "kind of transparency which is the opposite of secrecy" definition of openness [92], each step of research requires clear, accurate, and precise documentation. Comprehensive, clear, and accurate documentation is critical for replicability and reproducibility of research but is also critical for communicating to a larger audience than the research community and can encompass elements beyond those required to conduct the research. People can benefit from insights into the entire process, such as how and why a research question was formulated, what significance and impact answering the question could have, how the question relates to prior work, how the study was designed and executed, how the results were interpreted and presented, and what lessons were learned (92).

**Fig 2:**
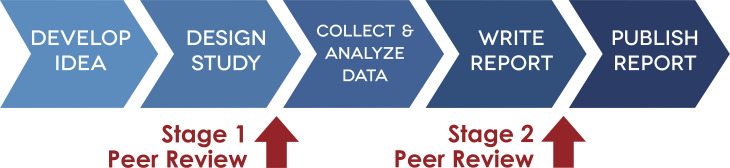
Each blue arrow in the diagram represents a research step that requires documentation. Each red arrow is an opportunity for a preprint. Image source: [157].

Prior to recruiting any participants and collecting any data, preregister your literature review, ethics statement [93], and methods. Preregistration can consist simply of documentation of plans for conducting a study, independent of peer review or a publisher, and can be submitted to an online preregistration site (for example osf.io, aspredicted.org, or the PROSPERO registry for systematic reviews at crd.york.ac.uk/prospero/). Preregistration can also involve submitting to a publisher to be "externally reviewed, and those that meet criteria will be accepted in principle prior to data collection" [94] (See Fig 2). A preregistration manuscript submitted for review and accepted in principle by a publisher is called a "registered report" [95,96]. This requirement for preregistration is not always optional, as human health research that involves a clinical trial typically requires preregistration of study plans (e.g. clinicaltrials.gov in the US, the WHO International Clinical Trials Registry Platform—ICTRP, as well as many other country-specific clinical trials registries across the globe).

Document any change or amendment as a project progresses. To make the documentation process easier, seek out established templates [97–101]. Strive toward reproducibility [74] (even for oneself in the future!) by providing self-contained, clear, and updated documentation and retaining data, code, recruitment documents, and other research artifacts to build upon in the future [102–104].

After submitting registered reports and articles for publication, post your articles to preprint servers such as bioRxiv or arXiv, to share your knowledge and stake your claims without waiting for the full publication cycle [105,106]. Publish raw materials of your research such as data, lab notebooks, and software in appropriate venues, such as data and methods journals, and in the trusted repositories mentioned above.

Document and publish often and in detail, including experimental designs and negative results, to receive feedback and detect and resolve errors early in the process [107]. Errors occur in public and in private, and while "making code and data open does not prevent errors, […] it does make it possible to detect them. […] People often worry that if they make their code and data open, errors will be found, but that is really the whole point: We need to make code and data open because this is how the errors can be found" [108]. The individuals who document the research don’t have to be the same people who conduct the research: assigning different people to document versus run a study encourages generally understandable documentation. Finally, link to your publications, shared data, and other documentation on your professional website, social media, and curriculum vitae (CV) [106]. Let colleagues know about innovative documentation practices you are trying.

## Rule 7: Publish and present accessibly

To best serve health research, communications at every stage of the research endeavor must be findable, accessible, interoperable, and reusable (FAIR; see Rule 5: make research reproducible) [84,85]. By accessible, we mean both easily retrievable and expressed in a manner that is clear and intelligible to the widest possible audience without unduly compromising the integrity of the information to be conveyed. This is a challenge not only because there are technical and abstract elements to any scientific study, but also because many scientists consider scientific journals as the sole conduit by which they convey results of their research.

When you have control over the license under which your work is published, choose a permissive license (e.g., [109,110]) and encourage consumers to use and share your work. Publish in open-access journals, being careful to choose appropriate, nonpredatory publications. Use checklists [111,112] to evaluate potential venues. Unless you must submit to a journal that disallows preprints, always submit your manuscript to a preprint server as well as a peer-reviewed journal. Tools like RoMEO (online, community-driven database of open-access policies) can help you navigate publisher licensing policies [113].

When you must publish under a closed license, deposit your article in a postpublication archive (eg, Hyper Articles en Ligne, HAL [114]) or on your own website once you are legally able. Some jurisdictions legally grant you the right to openly publish your closed license work after a specified embargo period; these laws may specify different embargo periods for different disciplines [115,116]. Some institutions (e.g., Harvard University, [117]) require open access for non-commercial use of their research. Consider making your work available in real-time on public platforms, such as Open Lab Notebooks, Open Science Framework, Labstep, GitHub, GitLab, Figshare, Zenodo, Dryad, protocols.io, and Aperture [104,118–126]. By making these products open and accessible, the scientific community will be able to build on your research more rapidly and more effectively.

Research publications and other informational websites are often dominated by a few languages, especially English. Translate your work and the work of others into different languages, and account for cultural and social factors; the French-language open-access publisher Science Afrique [127] is an example of a regionally focused effort. Create or update Wikipedia pages on published research findings [128,129], in multiple languages.

Strive to make research, not just your own, accessible to nonscientists and scientists alike. For broader dissemination, feedback, and engagement than traditional publishing venues provide, researchers should also consider publishing in social media, blogs, and other platforms as a project progresses [104,118–125]. Evidence indicates benefits to both data creators and the wider research community when research objects beyond books and articles are openly shared [70,130]. Even when submitting a manuscript to a traditional publisher, you can write a summary and/or a glossary of key terms [131], using language devoid of scientific jargon [132], add it as supplementary information to your manuscript, post it on your lab website, and share a link through social media to relevant groups. Consider using annotation tools [133–135] to make papers you are interested in accessible to a wider community. Demystify the scientific funding process by reporting research costs and citing successful examples of return on investment (ratio of benefit to cost).

Finally, in the future, accessibility will increasingly refer to machine readability for computer mining and interpretation of the literature. Placing data, metadata, and any other structured documentation into data repositories will make them more easily discovered, cited, and tracked by humans today and machines tomorrow. Permanent, versioned, and unique identifiers (such as DOIs) will make it easier for computers to help us more rapidly navigate and analyze the vast literature in the future.

## Rule 8: Emphasize research significance

Researchers all too often take for granted that the audience for their work is restricted to a narrow group of specialists who read and review their scientific articles and that the implications and significance of their work is readily apparent. However, because human health is a topic of immense interest, there will always be a great deal of attention on topics that relate to people’s hopes and concerns, especially by news media, and therefore there is a danger that the significance of a body of research will be misinterpreted. The onus is therefore often on researchers to communicate the meaning of their results and a clear context for their work and convey a strong sense of purpose and meaning that motivates an experiment’s design and drives any applications that are derived from the work.

For participants, let others know why you are participating in the research you participate in. For researchers, succinctly state the goals of each project, so that participants may understand not just their direct benefit, but how their contributions promote positive scientific research outcomes. Clearly publicize to all stakeholders the physical, realizable benefits of individual involvement in the research. Report on the implications of your research to wider audiences through traditional and nontraditional venues, from “news and views” pieces and press releases to Tweets, YouTube videos, and Science Cafe presentations.

For a researcher, the term “significance” confers an additional meaning distinct from importance and is referred to as “statistical significance,” Statistical significance is a commonly misunderstood and widely reported benchmark for believability of a study’s results. A critique of statistical significance reporting is beyond the scope of this editorial, but generally in statistical analyses, reporting a *p* value and using that value as a binary threshold is insufficient at best [1,5,90,91,136,137]. Thoroughly articulate statistical significance, including an explanation for both the selection of and practical interpretation of the statistical tests you performed in the context in which you performed those tests, the assumptions involved, and any alternative tests and assumptions that were considered but rejected. Put your research findings in context and communicate them clearly and cautiously and with appropriate caveats and considerations. Consider the relative size of the observed effects and consider and discuss not only the statistical but also the biological significance of your results.

## Rule 9: Advocate open principles

Practicing open science is best done not in isolation, but in a community of open science practitioners. This is never more true than in human health research, where health data can be difficult to collect, share, and analyze, and the research itself is most often done in silos. Coordinating the activities among people, the interoperability of methods, the sharing of data, and the inclusion of more diverse stakeholders is not only desirable, but essential. For open health research to be successful we must build such a community, and this is possible only if we strongly and persistently advocate for principles that underpin it. To assure our efforts are effective and genuine, we must identify and focus on priorities for advocacy. The Transparency and Openness Promotion (TOP) guidelines, released in 2015, provide community-driven standards for publishers and funders [138–140]. For individuals, promoting open health research can be as simple as initiating discussions in classrooms, conferences, and social events, and can be exercised in informal gatherings, such as dedicated Wikipedia editing sessions on open science topics, or open review sessions of articles on PREreview [141,142]. There are many steps that you can take to lead by example and promote the practice of open science today. We include some examples below from a list of recommendations we have curated [143].

Within your home institution: Catalyze open science practices through seminars, workshops, hackathons, and contests. Join groups that advocate evaluation or promotion criteria in support of open science. Pursue funding opportunities that require or permit open intellectual property. Opt for open methods rather than proprietary, licensed products. Apply liberal licenses to documents and software. Store data in free and open-access repositories.

In collaborations: Forge ties across labs to share resources. Collaborate with institutions that require open standards. Use collaborative software and collaborative software engineering practices. Publish a code of conduct for each project to clarify roles and help resolve disputes. Clarify contributor roles at the outset of a project to assign appropriate credit and accountability, especially for open contributions. Clarify when contributions to a project can be released. Avail yourself of experts in alternative and complementary methods to reduce bias, evaluate methods, and corroborate results. Participate in interdisciplinary open science and collaboration events.

In publications and presentations, publish in open-access venues and follow FAIR principles. Publish in open data and open methods journals. Follow community-supported data format and reporting guidelines. Insist on publishing experimental protocols and negative results. Boycott review or submission for publishers and publications that flout open standards. When reviewing others’ work, acknowledge attempts and provide recommendations toward more open science practices. Participate in open peer review, especially in languages other than English. Include an ethics section to articulate ethical considerations and implications. Make it clear where people can access open resources that you mention. When someone else mentions a resource, ask about access and usage restrictions. Include open resources on your webpage and CV.

## Rule 10: Take calculated risks

A variety of risks are inherent in research with human subjects, in communications that can influence health practices, and in open practices. Honest and open deliberation of these potential risks across the lifespan of the research is essential to trustworthy, impactful human health research. These various risks can arrive in isolation or combination and be known in advance or realized over time. As such, we should justify the decision of whether to assume these risks based on the ability to mitigate potential harms against benefits of knowledge gained.

Return on investment must be considered in choosing which risks to take, and some risks may be too costly even if the potential rewards are great [80,144]. Openness is a buzzword today, particularly in science, and as such openwashing ("to spin a product or company as open, although it is not" [145]) is both a practice to watch out for and an example of a risk that would be hard to justify in terms of value but easy to justify in terms of cost. Legal frameworks, particularly as relating to personal data and privacy, are a rapidly changing factor in assessing these risks. Consequently, cost-benefit analyses should be undertaken frequently. These analyses should be documented (see Rule 6: document everything) and shared (see Rule 8: emphasize research significance).

Acknowledge good-faith efforts that fail and encourage publication of negative results. Pushback against closed institutional traditions, challenge secretive practices [146], and explore nontraditional methods. Risks related to the other rules include going beyond accepted norms of ethical protections and partnership with nonscientists, systematically establishing greater clarity and accessibility of who does what and how for better appreciation, understanding, reproducibility, and advocacy.

Seek feedback from external stakeholders (i.e., target populations, funding agencies, local government and university officials) for your experimental design and methods before participating in or conducting an experiment; act on the feedback collected if deemed wise and not merely opinions or conventional wisdom. Also seek outside training for students and employees that includes options for nonacademic paths.

Seek interdisciplinary collaborations and spend a percentage of time and research effort working on projects outside your comfort zone. For example, have researchers spend 10% to 20% of their time on other projects of interest that they are passionate about. These can include topics of research that have received pushback in the field, deemed "too large to tackle," or those unlikely to produce confirmatory results but have the potential to incite new areas of research.

## Conclusion

We hope that the above list of simple rules is a helpful guide to follow best practices in open human health research. More importantly, we hope that you will use these as a starting point to address broken conventional practices of science and, where these rules fall short, share your own rules to improve the state of open, ethical, inclusive human health research. These rules are not comprehensive, but we are confident they capture many of the most salient, timely, and important principles that can guide open health research going forward. Be the change you seek in science [143] and strive to make human health research a more humane, effective, and, importantly, open endeavor.
